# Extraction and Characterization of Tunisian *Quercus ilex* Starch and Its Effect on Fermented Dairy Product Quality

**DOI:** 10.1155/2020/8868673

**Published:** 2020-08-04

**Authors:** Youkabed Zarroug, Mouna Boulares, Jamel Mejri, Bechir Slimi, Ghaith Hamdaoui, Saida Djebi, Fatma Saidi, Hanen Nasri, Dorra Terras Sfayhi, Mohamed Kharrat

**Affiliations:** ^1^Field Crops Laboratory, National Agronomic Research Institute of Tunisia (INRAT), Ariana, Tunisia; ^2^Research Unity Bio-preservation and Valorization of Agricultural Products UR13-AGR 02, Higher School of Food Industries (ESIAT), Tunis, Tunisia; ^3^Département de Génie Mécanique et Agro-Industriel, Ecole Supérieure des Ingénieurs de l'Equipement Rural (ESIER), Medjez el Bab, Tunisia; ^4^Laboratoire des Nanomatériaux et Systèmes pour les Énergies Renouvelables (LANSER), Centre de Recherches et des Technologies de l'Énergie Technopole Borj Cedria, BT 95, Hammam Lif 2050, Tunisia; ^5^Centre de Recherches et des Technologies de l'Energie (CRTEn) de Borj-Cédria, BP 901, Hammamlif 2050, Tunisia; ^6^High Institute of Technological Studies of Zaghouan, (ISET), Nabeul, Tunisia

## Abstract

In this study, a new starch has been isolated from acorn (*Quercus ilex*) fruits. The chemical composition of acorn flour showed its richness in carbohydrates (64.43%), proteins (8%), and fat (10%). The extraction yield of acorn starch was about 34.5%. Thus, the composition of extracted acorn starch and its physical and functional properties were studied. Acorn starch had high purity represented by low proportions of proteins (0.92%) and lipids (0.51%) with a pH of 5.3. The swelling power was 20.76 g/g, while the solubility was about 64.22% at 90°C which suggests that acorn starch has potential for use in food industries. The FT-IR spectra of isolated native starches have shown the main bands characterizing the starch. However, X-ray diffractograms exhibited an A- and B-type diffraction pattern. Furthermore, the effect of acorn starch incorporation at different levels (0.5%, 1%, 1.5%, and 2%) on the quality parameters of a fermented dairy product was investigated at the beginning of storage. The results demonstrated that the most suitable dose of acorn starch to be incorporated in the fermented dairy product was lower than 1%. This low concentration reduced syneresis, improved functional properties, and enhanced the viscosity of the fermented dairy product.

## 1. Introduction

Genus *Quercus* acorns belong to the family *Fagaceae,* which includes several species such as *Quercus robur*, *Quercus petraea, Quercus ilex*, and *Quercus pubescens* [1]. Due to the high variability of genus *Quercus* acorns, the chemical composition of this fruit varies with species and origin [2]. Being mainly a source of carbohydrates, fats, and fibers, acorn kernels are nutritionally comparable to many cereal grains. Moreover, these kernels contain proteins, high content of essential amino acids, vitamins (mostly A and C), and minerals. In addition, acorns are a good source of active compounds with an interesting antioxidant activity [1]. Generally, the caloric value of acorns ranges between 265 and 520 calories per 100 g [3]. However, acorns also contain a high concentration of tannic acid, a mild toxin giving them a bitter taste that can be removed by a variety of methods like soaking in water, boiling, or roasting [4].

On the other hand, generally, 31 to 55% of acorns mass were composed of starch [1]. Starch is a biodegradable carbohydrate polymer which has been widely studied due to its availability, price, and extensive use in food products and nonfood products [5]. In fact, starch is a raw material representing the principal component of many food formulations being responsible for important functional and textural properties and nutritional characteristics of the food products [5].

Additionally, today's consumers are demanding food products improving their health. Since acorns have always been an attractive food resource in human diet and are good sources of starch, these fruits show a large potential for commercial use, and thus, acorn flour may be an interesting component [4].

Yoghurt is one of the most popular fermented milk products widely consumed around the world as it provides nutritional and health benefits [6]. Yoghurt is fermented by the activity of *Streptococcus salivarius* ssp. *thermophilus* (*S. thermophilus*) and *Lactobacillus delbrueckii* ssp. *bulgaricus* (*L. bulgaricus*) [7].

Some studies focused on the improvement of yoghurt quality by reducing syneresis and enhancing viscosity and sensorial characteristics by adding whey proteins [8], fiber-rich fruit peel powder [9], seed mucilage or guar gum [10], exopolysaccharides [11], and hydrocolloids such as gelatin [12].

In Tunisian dairy industry, modified starch has been commonly used as a stabilizer, especially for many fermented dairy products in order to achieve desired viscosity and prevent syneresis [13]. Besides, there is no scientific research dealing with the effect of modified starch substitution by extracted acorn starch in dairy fermented products.

In this regard, in the present study, we evaluated the effect of acorn starch incorporation in milk in order to improve the physicochemical, organoleptic, and rheological quality as well as syneresis variation of a new produced fermented product after manufacture.

## 2. Materials and Methods

### 2.1. Sample Preparation

Acorns fruits belonging to the species *Quercus ilex* were manually collected from Ayn Darahim region of Jendouba in the northwest of Tunisia. Then, kernels were hand-peeled, dried at 40°C for 3 days, and then milled into flour. The acorn flour was stored in glass flasks at about 4°C for further starch extraction and analysis.

### 2.2. Starch Extraction

Acorn starch was extracted as reported in [14]. Acorn flour was dispersed in 0.3% sodium hydroxide (1 : 5, w/w), well mixed, and allowed to stand for 2 h. The dispersion was then filtered using a mesh sieve. The starch solution was allowed to settle for 24 h, and the supernatant was subsequently decanted and discarded. The precipitate containing the starch was rinsed several times with distilled water, air-dried, and stored in sealed containers for further analysis. The starch yield was calculated using the following equation:

(1) $$\begin{array}{c} \text{starch yield}\left(\%\right)=\frac{\text{starch extracted }\left(\text{g}\right)}{\text{acorn flour }\left(\text{g}\right)}\times 100. \end{array}$$

### 2.3. Starch Analysis

#### 2.3.1. Chemical Composition

Chemical composition of acorn flour and extracted starch was determined according to the AOAC methods for moisture, ash, protein, lipid, and carbohydrate contents [15]. The pH of acorn starch was determined using the method described in [16]. Objective color of different samples was measured with a colorimeter (model CR-300, Konica Minolta Sensing Inc., Tokyo, Japan) recording *L*^∗^ (a measure of lightness, ranging from 0 (black) to 100 (white)), *a*^∗^ (ranging from −100 (greenness) to +100 (redness)), and *b*^∗^ (ranging from −100 (blueness) to +100 (yellowness)).

#### 2.3.2. Solubility, Swelling Power, and Water Absorption Capacity

Solubility, water absorption, and swelling power patterns at 60, 70, 80, and 90°C were determined according to the modified method described in [5]. Briefly, 40 ml of a 1% starch suspension (w/v) was prepared in preweighed centrifuge tubes. A magnetic agitator was placed into the tube, and it was kept at a constant temperature (60, 70, 80, or 90 °C) in a water bath for 30 min. The suspension was then centrifuged at 2500 rpm for 15 min, and the supernatant was decanted. The swollen granules were dried at 50°C/25 min in a hot air oven and were reweighed. Water absorption capacity (g/g) was expressed as the weight of the gel formed per sample divided by treated sample weight. For the determination of solubility and swelling power, 10 ml from the supernatant was dried in an air convection oven at 120°C for 4 h until constant weight. The solubility and swelling power were calculated with equations (2) and (3), respectively:

(2) $$\begin{array}{c} \%\text{ solubility}=\frac{\text{weight of solid solubles in supernatant}\left(\text{g}\right)}{\text{weight of the sample }\left(\text{g}\right)}\times 100, \end{array}$$

(3) $$\begin{array}{c} \%\text{ swelling power}=\frac{\text{weight of gel }\left(\text{g}\right)}{\text{weight of the sample }\left(\text{g}\right)-\text{weight of solid soluble }\left(\text{g}\right)}\times 100. \end{array}$$

#### 2.3.3. Refrigeration and Freezing Stability

To evaluate the stability of acorn starch under refrigeration and freezing, 400 ml of 6% starch suspension was heated to 95°C at a rate of 1.5°C/min. Then, the suspension was held at this temperature for 15 min, cooled to 50°C at the same rate, and held at this second temperature for another 15 min. Portions of 50 ml were placed in centrifuge tubes, cooled down to room temperature, and stored at 4°C and −20°C. These tubes were centrifuged at 4000 rpm for 10 min. Measurements of water separation from the starch gels at 24, 48, 72, and 96 h were taken [5]. The syneresis rate was calculated using the following equation:

(4) $$\begin{array}{c} \text{syneresis }\left(\text{\%}\right)=\;\frac{\text{weight of syneresis water }\left(\text{g}\right)}{\text{weight of gel}\left(\text{g}\right)\;}\times 100. \end{array}$$

#### 2.3.4. Fourier Transform Infrared Spectroscopy

All spectra were collected using a PerkinElmer Spectrum single-bounce ATR accessory with a diamond crystal. Dry starches were equilibrated at laboratory humidity (50% RH) and were dispersed in a matrix of KBr at room temperature, and the mixture was then pressed into pellets. The range of spectra was from 400 to 4000 cm^−1^ with a spectral resolution of 4 cm^−1^.

#### 2.3.5. X-Ray Diffraction

X-ray diffraction (XRD, Bruker D8 Advance) in the Bragg–Brentano configuration using Ni-filtered Cu K*α* radiation (*k* = 1.5418 Å) operated at 40 kV and 40 mA was used to determine the degree of starch crystallinity using the method reported in [17]. Diffractograms were obtained in 2 h with a scanning speed of 2°/min and a scanning step of 0.02° was used.

### 2.4. Effect of Acorn Starch Incorporation on a Fermented Dairy Product Quality

#### 2.4.1. Fermented Dairy Product Preparation

The fermented dairy product was manufactured from raw milk in the Tunisian SLD industry (industry of dairy products). Milk (16°D acidity, 1027.5 density, 6.61 pH, and 18 g/l fat content) was homogenized, pasteurized at 95°C for 5 min, and cooled down to 45°C. After that, three batches of pasteurized milk were prepared and each batch was incorporated with one concentration of acorn starch (0%, 1%, and 2%) and inoculated with a freeze-dried starter culture, consisting of a combination of *Streptococcus salivarius subs. thermophilus* and *Lactobacillus delbrueckii subs. bulgaricus*. Control (0% acorn starch) and enriched batches were incubated at 45°C until the gel structure was formed and pH reached 4.8. Then, fermented dairy products were stirred, distributed in flasks, and stored at 4°C. Finally, sampling of the resulting fermented dairy product was performed on the first day of storage at 4°C. All determinations of physicochemical, technological, and nutritional characteristics of produced products were done in triplicate in order to choose the best added concentration of acorn starch improving this fermented dairy product quality.

#### 2.4.2. Physicochemical Characteristics and Syneresis

Total solids of fermented dairy samples were determined at 90°C using an infrared dryer. The pH of the prepared samples was measured with a pH meter. The titratable acidity was (expressed as g lactic acid 100 mL^−1^) determined by the potentiometric method according to the IDF standard [18]. Syneresis was measured by centrifugation technique according to Celik et al. [19], with several modifications. The color was measured using a colorimeter as reported in [20].

#### 2.4.3. Rheological Measurements

Rheological properties were determined with a rheometer (proRheo R180) using a cone-plate geometry (60 mm in diameter). Fermented dairy samples were carefully placed in the measuring system, left to rest for 30 min for structure recovery, and analyzed to determine viscosity [21].

#### 2.4.4. Microbiological Analysis

Microbiological analyses of control and enriched fermented dairy product were performed to determine the influence of acorn starch addition on the viability of starter and total bacterial count during the refrigerated storage period. The total mesophilic bacteria were enumerated by using Plate Count Agar Medium (Oxoid, Ltd., Basingstoke, England), *Streptococci* were counted on M17 (Biokar Diagnostics, Beauvais, France) agar, and *Lactobacilli* were counted on MRS agar (Biokar Diagnostics, Beauvais, France). Yeasts, molds, and coliforms were enumerated according to the methods described in [22]. The samples were analyzed in duplicate.

### 2.5. Statistical Analysis

All tests were possessed in three replications, and the average values were performed by variance analysis (ANOVA) using STATISTICA software. Duncan's multiple range tests were used at the significance level of 5% to highlight significant differences among the fermented dairy samples.

## 3. Results and Discussion

### 3.1. Characterization of Acorn Starch

#### 3.1.1. Chemical Composition of Acorn Flour and Starch

The chemical composition of acorn flour and acorn starch extracted from *Quercus ilex* species is shown in Table 1. Carbohydrates, protein, ash, and moisture contents of acorn flour were 64.43%, 8%, 1.62%, and 15.95%, respectively. These results were partially in accordance with those reported by Charef et al. [23] and Masmoudi et al. [24]. Interestingly, Tunisian acorn flour was found also to contain significant content of fat (10%), which was within that reported by Charef et al. [23] on Algerian acorn, but higher than that found in *Quercus lobata* (4.25%) and *Quercus shrubs* (4.05%) [25]. However, this content was lower than that (14.30%) registered by Masmoudi et al. [24], on *Quercus suber.* L. In the literature, acorn fat content varies from lower than 2 to 30% [24] and this variation could be attributed to many factors such as plant varieties, cultivation climate, ripening stage, and the extraction method used. These findings reveal the valuable potencies of *Quercus ilex* acorn kernels.

Since *Quercus ilex* acorn flour contains a high amount of carbohydrates, it may constitute a valuable source of starch which can be used in human food. Concerning starch, we noted that the yield of the extraction was approximately 34.5%. This result was in disagreement with that (63%) found by Masmoudi et al. [24] on *Quercus suber.* L species but slightly similar to that registered by Nand et al. [25] for *Manihot esculenta* (cassava) (32.10%). However, the obtained yield was higher than those reported, respectively, from *D. pyrifolia* tubers (26.64%) [25] and *D. hispida* (11.42%) [16]. The richness of acorn fruit is very interesting in food industry. In fact, it can be used as an alternative raw material for flour production or as an ingredient in food products, such as bread and pastry [1] and particularly products for consumers with celiac disease [26]. Besides, the content of starch in acorn flour gave it good functional characteristics related to starch such as viscosity, swelling, and gelling [24].

After extraction, we observed that the moisture content of acorn starch under study was about 10.17%. This low moisture content of the acorn starch, less than 20%, was acceptable for commercial starch as it was recommended for safe storage with moisture content less than 13% as reported by Pérez-Pachecoa et al. [5]. Besides, acorn starch presented low proportions of proteins (0.92 ± 0.01%) and lipids (0.51 ± 0.05%), which was in accordance with the findings on Ramon and corn starches [5]. For ash content (2.66%), we registered higher values than those obtained on Ramon (0.47%) and corn (0.02%) starches. However, the low protein, fat, and ash content show a high purity and quality of the extracted acorn starch. The pH of the acorn starch was approximately 5.3 reflecting the acidic conditions of the extracted starch. This pH value was higher than that of *D. pyrifolia* (3.43) [25] and *D. hispida* (4.48) [16] but lower than that of *Solanum tuberosum* (potato) (6.22).

Acorn starch exhibited a slightly yellow-white color while acorn flour was darker. Indeed, extracted acorn starch showed a high lightness *L*^∗^ value (85.03) compared to the acorn flour (51.13). For acorn starch, the obtained values of *a*^∗^ (0.52) and *b*^∗^ (10.2) were different from those found in acorn flour (2.94 and 14.67, respectively). These results indicated that acorn starch presents yellow tones. In the present study, the results found for color parameters were similar to those reported on Ramon starch [5]. These findings showed that acorn fruit is a good source of starch that can be used in food industry without the necessity of chemical or genetic modifications [24]. This polysaccharide may be industrially applied as emulsifiers, stabilizers, and thickeners in food and also as prebiotic growth promoter [26].

#### 3.1.2. Swelling Power, Solubility, and Water Absorption of Acorn Starch

**Figure 1:**
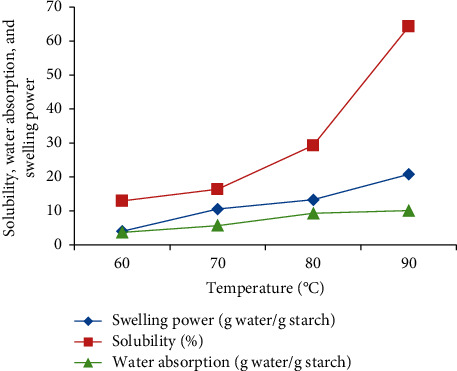
Swelling power, solubility, and water absorption of acorn starch.

As shown in Figure 1, swelling power, solubility, and water absorption values of extracted acorn starch increased gradually with the increase of temperature from 60°C to 90°C. The swelling power values (4–20.76 g/g) are low compared to those of other starch sources such as potato starch (56.2–64.7 g/g) [27], but comparable to chestnut starches (13.6–17.3 g/g) [28]. The lower observed values for swelling power could be due to the amylose content of the acorn starch [29], starch molecule's ability to hold water, hydrogen bonding, and the degree of crystallinity [30].

The solubility of the extracted acorn starch had significantly increased at 80°C–90°C to reach a value of 62%. This solubility of the extracted acorn starch increased significantly from 12.95% at 60°C to a value of 64.22% at 90°C. These values were higher than those registered on starches from *D. pyrifolia, Dioscorea opposite, D. alata*, *D. nipponica*, *D. bulbifera*, *and D. septemloba* [31]. The high solubility of acorn starch at a lower temperature in water encourages us to use this starch as an additive in fermented yoghurt and milk products.

For the water absorption capacity of starch, it corresponds to the hydrogen bonding between water molecules and hydroxyl groups in the starch molecules and starch chains as well as diversification of the starch granule structures [32].

#### 3.1.3. Refrigeration and Freezing Stability of Acorn Starch

The stability variation of the extracted acorn starch under refrigeration and freezing conditions during time is shown in Table 2. In general, to evaluate the stability of starch during storage, it is necessary to verify the expulsion of water (syneresis) contained in gels as a consequence of the reorganization of starch molecules [33]. Results showed that the syneresis of acorn starch increased under refrigeration (4°C) and freezing (−20°C) conditions with storage time increase. According to the literature, starches with high amylose content such as potato (20.1–31.0%), maize (22.4–32.5%), taro (28.7–29.9%), and cassava (18.6–23.6%) present high syneresis, due to the large amount of water expelled during the retrograding process [5].

#### 3.1.4. X-Ray Diffraction and FT-IR Spectra

**Figure 2:**
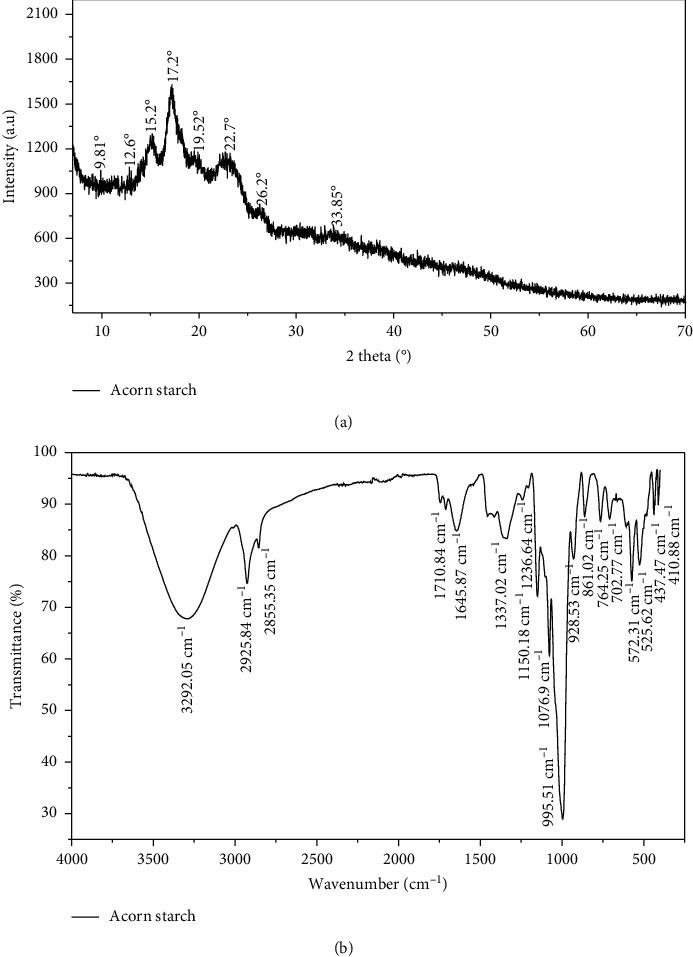
X-ray diffraction pattern (a) and FT-IR spectra (b) of acorn starch.

The X-ray diffraction and the FT-IR spectra of acorn starch are shown in Figures 2(a) and 2(b).  Figure 2(a) showing the X-ray diffractogram of the extracted acorn starch can explain the classification of the studied starch granules as the A-, B-, or C-type. In general, cereal starches have an A-type pattern, whereas tuber starches display the B-type pattern, and certain roots and legumes starches show a C-type pattern [11]. The obtained X-ray diffractogram shows four intense diffraction peaks at 15.2°, 17.2°, 19.52°, and 22.7° of 2ϴ. The strong reflections at 15.2° and 22.7° of 2*θ* were classified as the A-type pattern, which characterized most cereal starches [34]. However, the peaks at approximately 17.2° and 19.52° of 2*θ* were characterized as the B-type pattern. Our results were similar to that reported for starch from *Quercus glandulifera* Bl. [35] and *Dioscorea pyrifolia* tubers [27]. Since the X-ray diffraction measurement system ensures the error range of 2*θ* ≤ ±0.02, the observed peak shifts are not experimental error, which is in good agreement with the previously reported phase.

The FT-IR spectra of acorn starch in the range of 400 to 4000 cm^−1^ are shown in Figure 2(b). Our results showed the presence of a large band with a maximal absorption at about 3292.05 cm^−1^ which indicated the stretch vibrations of O–H groups of the starch molecules. Besides, two second bands were observed at 2925.84 and 2855.35 cm^−1^, probably resulting from the stretching of C–H bonds. The amide group of proteins band was found between 1645.87 and 1710.84 cm^−1^ reflecting the C–O stretching. The observed peaks at 1645.87 cm^−1^ were also assigned to water molecules absorbed in the amorphous region [36]. The region located from 410.88 to 1150.18 cm^−1^ originated mainly from carbohydrate vibrations. In fact, the bands at 1150.18 and 1076.9 cm^−1^ observed in the spectra of acorn starch were attributed to the presence of *β*-glycoside and glucoside and the stretching of the C–O bonds. However, the observed band at 995.51 cm^−1^ was characterized by partially crystalline materials [37].

### 3.2. Effect of Acorn Starch Incorporation on Fermented Dairy Product Quality

In order to choose the best concentration of extracted acorn starch to be incorporated later in a fermented dairy product at industrial scale, biochemical, physical, rheological, and color properties, variations were observed as a function of starch dose variation at the first day of storage. As shown in Table 3, all parameters of the fermented dairy product enriched with acorn starch at different concentrations differed from those of the control product.

#### 3.2.1. pH and Dornic Acidity Variations

The initial composition of all tested fermented dairy products is shown in Table 3. On the first day of storage, pH values reached about 4.6 ± 0.07 and 4.59 ± 0.01 after refrigeration for control and fermented dairy products made with 1% of acorn starch. These values were higher than that noted on the fermented product enriched with 0.5% of acorn starch, suggesting that the pH decrease in this sample was mainly due to a better lactic acid production as reported by Altemimi [36]. We observed that pH increased with higher starch doses to reach the values of 4.8 ± 0.00 and 5.00 ± 0.14 in the fermented product prepared, respectively, with 1.5% and 2% acorn starch. Similar results showed a pH increase in yoghurt fortified with fibers [7, 9]. In this connection, we noted similar (*P* < 0.05) initial Dornic acidity values observed on control and yoghurt enriched with 1% of acorn starch. For higher acorn starch doses, we registered a decrease of acidity. This result may be due to the fact that adding high concentrations of extracted starch could reduce the amount of water which makes it difficult for starter culture to metabolize lactose sugar, and thereby, the amount of produced lactic acid was reduced [38]. Besides, our finding for low concentrations of added acorn starch was in agreement with the results reported by Vinha et al. [26], suggesting that this polysaccharide may be used in food industry as prebiotic growth promoter. In fact, a significant relationship was proved between the gradual increase in acidity of yoghurt and the amount of lactic acid produced as the result of lactose fermentation by the associative growth of the two thermophilic, homofermentative lactic acid bacteria [39].

#### 3.2.2. Total Solids Variation

Results showed that initial dry matter levels were between 19.41 ± 1.68% and 28.5 ± 0.70% for all tested fermented dairy products (Table 3). The total solids content increased with acorn starch concentrations lower than 1%, and no significant differences (*P* > 0.05) were noted between these two enriched fermented dairy products and the control. However, total solids values decreased with higher doses to reach the lowest value for 2% added acorn starch fermented dairy product. Hence, the presence of acorn starch at high concentration decreased the total solids content of milk bases.

#### 3.2.3. Viscosity Variation

For consumer products, texture is an important parameter of the quality that makes an impact on the pleasure of eating. In this study, as shown in Table 3, we observed that the highest viscosity value noted on the fermented dairy product made with 1% of acorn starch was about 1.73 ± 0.33 Pa.s^n^. This concentration was shown able to improve the rates of aggregation and curd firming reactions in the casein gels. Moreover, the results revealed that the high concentration (2%) of acorn starch gives rise to the decrease of consistency coefficients (0.69 ± 0.01 Pa.s^n^). This result can be explained by the fact that the higher polysaccharide content resulted in the formation of polysaccharide-protein interactions that are weaker than protein-protein bonds [40] leading to viscosity reduction in fermented dairy samples containing acorn starch.

Also, this finding can be explained by the breakdown of the fermented dairy product structure after manufacture and the decrease of the total solid content in enriched fermented dairy product, especially when acorn starch concentration exceeds 1% (*P* < 0.05).

#### 3.2.4. Syneresis Variation

In this study, the addition of acorn starch was associated with a decrease in viscosity and an increase in syneresis. At the beginning of storage, the use of 0.5% and 1% of acorn starch was associated with weak whey separation. Our result did not show a significant difference (*P* > 0.05) between control and these two enriched fermented dairy products. These findings could be related to total solids. In fact, when dry extracts increased (fermented dairy product samples with 0.5% and 1% acorn starch), syneresis decreased as reported in [39].

Generally, we observed high serum exudation with the increase of acorn starch incorporation with registered values of 34.6 ± 0.85 and 36.9 ± 1.27, respectively, for the 1.5% and 2% acorn starch added fermented dairy products. The protein network generally governs these rheological and physical properties of yoghurt. In fact, hydroxyl groups of amylose and amylopectin bind water and thus increase the viscosity [41]. Consequently, the competition of other molecules such as proteins and exopolysaccharides may affect the swelling process of starch granules and alter their functional properties, for example, the reduction of serum retention as observed in this study.

#### 3.2.5. Color Parameter Variations

When evaluating color parameters, *L*^*∗*^, *a*^*∗*^, and *b*^*∗*^ values indicated that starch incorporation decreased the fermented dairy product lightness and redness and increased yellowness. The results of color parameters of fortified and control samples are displayed in Table 3. In this study, statistical analysis revealed a significant difference (*P* < 0.05) between all samples in *L*^*∗*^ (lightness). This finding was in agreement with those reported in [42], suggesting that the presence of caseins leads to a natural opacity of milk during storage. Also, we noted a significant difference (*P* < 0.05) on the first day of storage between control and fortified samples for *a*^*∗*^ (red color) and *b*^*∗*^ (*P* > 0.05) (yellow color) between the different obtained fermented dairy products. This result was due to the yellow color of added starch which may affect the aspect and the final color of the product, during processing. In the light of these findings, we can conclude, as shown by Masmoudi et al. [24] on acorn flour, that acorn starch could be applied in food industry due to its good technological properties and as a natural colorant in food formulations based on starch.

#### 3.2.6. Microbiological Property Variations

In this study, the findings corresponding to the enumeration of yeasts, molds, and total and fecal coliforms obtained on control and enriched fermented dairy products showed the absence of this flora at the beginning of storage (data not shown). Hence, our fermented dairy product enriched with different acorn starch concentrations had a satisfactory hygienic quality.

## 4. Conclusion

The biochemical quality of acorn flour, a nonconventional source, showed its richness in carbohydrates and proteins. Acorn produced a starch yield of 35.4%. Thus, the functional and chemical properties of extracted starch from acorn fruit demonstrated that it can be used as an ingredient in food industries requiring high processing temperatures. The results of this study showed that addition of acorn starch during manufacturing of a fermented dairy product showed a slight distinction compared to the control sample. In light of these reports, it can be concluded that acorn starch dose lower than 1% was able to improve or maintain the characteristics of conventional fermented dairy product, but this result has to be examined further during the refrigerated storage.

## Figures and Tables

**Table 1:** Chemical composition of acorn flour and acorn starch.

Components	Acorn flour	Acorn starch
Yield (%)	86 ± 0.13	34.5 ± 0.01
Moisture (%)	15.95 ± 0.02	10.17 ± 0.09
Fat (%)	10 ± 0.03	0.51 ± 0.05
Protein (%)	8 ± 0.05	0.92 ± 0.01
Ash (%)	1.62 ± 0.05	2.66 ± 0.01
Carbohydrates (%)	64.43 ± 0.01	—
pH	—	5.3 ± 0.01
*L* ^∗^	51.13 ± 0.02	85.03 ± 0.09
*a* ^∗^	2.94 ± 0.03	0.52 ± 0.01
*b* ^∗^	14.67 ± 0.01	10.2 ± 0.15

**Table 2:** Refrigeration and freezing stability of acorn starch.

Time (h)	Syneresis to refrigeration 4°C (%)	Syneresis to freezing −20°C (%)
24	42 ± 0.02	26 ± 0.01
48	49.34 ± 0.51	36.18 ± 0.42
72	53.3 ± 0.02	38.47 ± 0.01
96	55.57 ± 0.01	41.43 ± 0.01

**Table 3:** Chemical, physical, and rheological parameters of control and enriched fermented dairy products with different concentrations of acorn starch on the first day of storage.

Composition	Acorn starch (%)				
	0 (control)	0.5	1	1.5	2
pH	4.6 ± 0.07a	4.54 ± 0.06a	4.59 ± 0.01a	4.8 ± 0.00b	5.00 ± 0.14c
Acidity (°D)	83 ± 0.00b	84.5 ± 0.70b	83.5 ± 0.70b	81 ± 1.41a	79.5 ± 0.70a
Total solids (%)	26.95 ± 1.77c	28.5 ± 0.70c	27.45 ± 2.05c	23.7 ± 0.42b	19.41 ± 1.68a
Viscosity (Ps.s^n^)	1.6 ± 0.07cd	1.25 ± 0.21bc	1.73 ± 0.33d	0.89 ± 0.04ab	0.69 ± 0.01a
Syneresis	32.4 ± 0.57a	32.75 ± 1.06a	32.15 ± 0.92a	34,6 ± 0.85ab	36.9 ± 1.27b
Luminosity L^*∗*^	37.13 ± 0.46c	33.76 ± 1.19b	33.38 ± 1.02ab	31.64 ± 0.11d	27.36 ± 0.65a
Red color *a*^*∗*^	−0.43 ± 0.11a	−1.10 ± 0.02ab	−1.24 ± 0.03b	−2.03 ± 0.08c	−2.62 ± 0.11d
Yellow color *b*^*∗*^	16.95 ± 0.58a	19.52 ± 0.57b	22.4 ± 0.10c	22.42 ± 0.40cd	24.02 ± 0.84d

Values with different letters are significantly different *P* < 0.05.

## Data Availability

No data were used to support this study.
